# High-density scalp electroencephalogram dataset during sensorimotor rhythm-based brain-computer interfacing

**DOI:** 10.1038/s41597-023-02260-6

**Published:** 2023-06-15

**Authors:** Seitaro Iwama, Masumi Morishige, Midori Kodama, Yoshikazu Takahashi, Ryotaro Hirose, Junichi Ushiba

**Affiliations:** 1 Department of Biosciences and Informatics, Faculty of Science and Technology, Keio University, Tokyo, Kanagawa Japan; 2 Graduate School of Science and Technology, Keio University, Tokyo, Kanagawa Japan

**Keywords:** Brain-machine interface, Biomedical engineering, Data publication and archiving

## Abstract

Real-time functional imaging of human neural activity and its closed-loop feedback enable voluntary control of targeted brain regions. In particular, a brain-computer interface (BCI), a direct bridge of neural activities and machine actuation is one promising clinical application of neurofeedback. Although a variety of studies reported successful self-regulation of motor cortical activities probed by scalp electroencephalogram (EEG), it remains unclear how neurophysiological, experimental conditions or BCI designs influence variability in BCI learning. Here, we provide the EEG data during using BCIs based on sensorimotor rhythm (SMR), consisting of 4 separate datasets. All EEG data were acquired with a high-density scalp EEG setup containing 128 channels covering the whole head. All participants were instructed to perform motor imagery of right-hand movement as the strategy to control BCIs based on the task-related power attenuation of SMR magnitude, that is event-related desynchronization. This dataset would allow researchers to explore the potential source of variability in BCI learning efficiency and facilitate follow-up studies to test the explicit hypotheses explored by the dataset.

## Background & Summary

Closed-loop neurofeedback using non-invasive neural recordings has been an attractive approach for neurogenic motor diseases and mood disorders, inducing targeted neuroplastic changes associated with aberrant behavior^1–8^. Through manipulation of the targeted neural activities or their covariation, specific brain functions can be dynamically improved. For instance, the neurofeedback training of human motor cortical activities modulates the motor control ability in healthy populations and patients with movement-related disorders^8–11^.

The clinical application of neurofeedback training combined with robotic devices is promising for motor function, especially post-stroke hemiplegia^5,8,12–16^. To train voluntary control of neural activities the timing of finger movement can be controlled via finger orthosis with motors dependent on the intrinsic changes in the motor cortex activities using scalp electroencephalograms (EEG) for online extraction of corticomotor excitability. The brain-state dependent control of external devices can be neurofeedback training when patients were instructed to self-regulate sensorimotor activity by performing motor imagery of paralyzed fingers^17,18^. The repetitive use of the closed-loop system between the human brain and the computer, namely brain-computer interfaces (BCIs) leads to systematic improvement of upper limb motor function putatively by inducing neuroplastic changes in the neural circuitries that survived stoke^12,19–21^.

In the established BCI-based neurofeedback training protocol, the sensorimotor rhythm (SMR) component act as the representation of the human motor cortical excitability^22–26^. Basic neuroscience research has revealed that SMR power attenuation, captured by the EEG spectral power in 8–30 Hz derived from central areas reflects the event-related desynchronization (ERD) of populational neural activities in the primary motor cortex (M1)^27–29^. Thus, repetitive practice of voluntary control of SMR-ERD leads to successful motor recovery after participants learned to control SMR-based BCI through neurofeedback training^5,6,8,13,18^.

However, there is substantial variability in the learning efficacy for self-regulation of SMR-ERD, even for healthy participants. A variety of research have explored the source of the variability; the neurophysiological interaction with other cortical regions^30–32^ or cortico-subcortical loop^33^, intrinsic neural oscillations^34^, or even experimental conditions such as the sex of exprimenter^35^. Moreover, some researchers have concluded that there are a number of participants termed BCI illiteracy in a part of the population, who is unable to learn BCI control even after extensive training^36–38^. Collectively, there remains unclear the critical characteristics of participants that influence the ability to learn self-regulation of neural activities.

Here, to enable the comprehensive analysis of the variability in BCI performance, we provide the EEG data of BCI-based neurofeedback training of SMR-ERD control, in total more than 130 participants. The EEG setup employed in all studies was a 128-channel high-density EEG cap, making it possible to assess the neurophysiological properties outside the common BCI target region, which is the SM1 contralateral to the imagined hand. Moreover, the experimental conditions, such as the trial duration, total amount of training, and the object employed for the neurofeedback training are different across the dataset. Therefore the integrated analysis of these datasets would allow us to test the hypotheses regarding the factor behind the variability of neurofeedback efficacy.

## Methods

All experiments to collect the datasets were performed in accordance with the Declaration of Helsinki and the experimental procedures were approved by the Ethics Committee of the Faculty of Science and Technology, Keio University (IRB approval number: 2020–38 or 2021–74). Written informed consent to participate in the present study was obtained from every participant.

The four datasets provided in the present collection used the same experimental setup to collect the high-density scalp EEG during BCI-based neurofeedback. EEG signals were measured at a 1000 Hz sampling rate and electrodes were placed according to the international 10–10 electrode positions using the HydroCel Geodesic Sensor Net (EGI, Eugene, USA). The EEG signals were then amplified and digitized with the GES 400 (EGI, Eugene, USA). Cz and CPz channels were set as the reference and ground channel, respectively^39^. Participants were seated in a comfortable chair and instructions related to the experiments and neurofeedback were provided through the display in front of the participants.

**Fig. 1:**
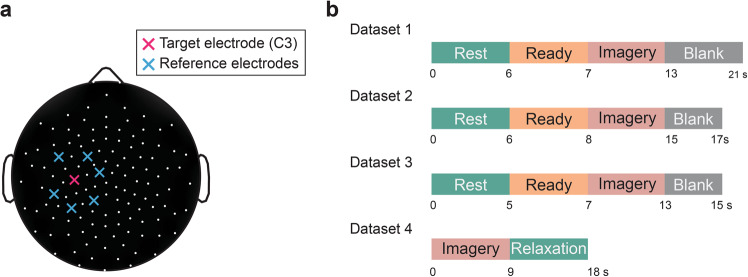
Experimental configuration. (**a**) Electrode locations used for online BCI operation. The pink cross indicates the electrode around the sensorimotor cortex contralateral to the right hand (C3 channel in the international 10–20 system) and blue crosses indicate the reference electrode used for the large-Laplacian filter. (**b**) Time-courses of a trials used in each dataset.

The exact experimental procedures and the analytic pipelines for the online calculation of SMR-ERD magnitude were variable. However, the concept for the signal processing was in common; since SMR-ERD derived from the contralateral SM1 reflects the corticomotor excitability^23–26,40^, the targeted region for the neurofeedback was selected as the relative spectral power change in 8–13 Hz derived from C3 electrode (The pink electrode in Fig. 1a). In the experiment to collect Dataset 1, 2 and 3, a fixed frequency band (alpha: 8–13 Hz) was used to calculate SMR-ERD magnitude. A large Laplacian spatial filter was applied to extract the localized activity^41^. The electrode placement used for the spatial filtering was shown in the Fig. 1a. In the experiment to collect Dataset 2, in addition to the large Laplacian filter, artifact subspace reconstruction algorithm was applied^42^. In the experiment to collect Dataset 4, a bivariate BCI employing the alpha-band activity and beta-band (14–30 Hz) was used. The frequency bands were adjusted to the participant-specific response frequency. The detailed protocol was described elsewhere^43^.

Time courses of trials in each dataset was described in the Fig. 1b. During experiment, participants were instructed to perform kinesthetic motor imagery of right finger movement during imagery period, and to keep eyes opened and avoid from excessive body move during rest period. A blank period was served to allow participants to blink and move body and take a break.

For each experiment, different numbers of blocks and trials were adopted. These differences in the experimental design may be one source of the heterogeneity of learning efficacy. In the experiment for Dataset 1, 30 participants (25 males and 5 females, age 21.23 ± 2.2, all right-handed) underwent 6 blocks of neurofeedback training for consecutive two days. Each block consists of 20 trials with a 6-second resting period, a 1-second ready period, a 6-second imagery period and a 8-second blank period (Fig. 1b). During the blank period, participants received a monetary reward (increase in the total amount of money) or punishment (decrease in the total amount of money), or neutral (no monetary feedback). Specifically, for the reward group, the existence of the additional fee for the participation of experiment was informed before the experiment and the fee was added based on the trial-by-trial performance of the BCI sessions. Meanwhile, for the punishment group, the amount of fee for the participation of experiment was introduced before the experiment and the fee was subtracted based on performance. In addition, the pre-and post-evaluation block was set to assess the offline effect of neurofeedback training; participants performed 20 trials with the time course identical to the training block without the neurofeedback. Neurofeedback of SMR-ERD magnitude was provided as the height of the bar during the imagery period.

In the experiment for Dataset 2, 30 participants (28 males and 2 female,s age 20.97 ± 1.1, all right-handed) underwent 6 blocks of neurofeedback training in a single day. Each block consists of 20 trials with a 6-second resting period, a 2-second ready period, a 7-second imagery period and a 2-second blank period (Fig. 1b). In addition, the pre- and post-evaluation block was set to assess offline effect of neurofeedback training; participants performed 20 trials with the time course identical to the training block without neurofeedback. During the training blocks, neurofeedback of SMR-ERD magnitude (8–13 Hz) was provided during the imagery period. Participants were randomly allocated to one of three feedback types: bar height, hand movement, or no feedback.

In the experiment for Dataset 3, 22 participants (21 males and 1 female, age 21.04 ± 0.7, all right-handed) underwent 15 blocks of neurofeedback training in a single day. Each block consists of 10 trials with a 5-second resting period, a 2-second ready period, a 6-second imagery period and a 2-second blank period (Fig. 1b). In addition, for every 5 blocks of training, an evaluation block was set to assess the offline effect of neurofeedback training; participants performed 10 trials with the time course identical to the training block without the neurofeedback. Neurofeedback of SMR-ERD magnitude was provided during the rest and imagery period. Participants were randomly allocated to one of two feedback types: gamified tail-like object or an abstract speedometer and all participants underwent the evaluation sessions. In the neurofeedback conditions participants were instructed to move the object bidirectionally to the target presented on the left side during the rest period and right during the imagery period, respectively. Participants were instructed that the object moves to left when they are successfully relaxed during the rest period and to right when they successfully performed kinesthetic motor imagery of right finger. The maximum range of SMR-ERD is normalized based on the SMR-ERD magnitude during the previous evaluation block.

In the experiment for Dataset 4, 56 participants (49 males and 7 females, age 23.67 ± 7.5, 55 right-handed and 1 left-handed) underwent 3 blocks of neurofeedback training in a single day. Each block consists of 30 trials with a 9-second imagery period, and a 9-second relaxation period (Fig. 1b). During the relaxation period, participants were instructed to actively relax their right finger which was attempted to move during the imagery period. In addition, the pre-and post-evaluation block was set to assess the offline effect of neurofeedback training; participants performed 20 trials with the time course identical to the training block without the neurofeedback. Neurofeedback of SMR-ERD magnitude was provided during the relaxation and imagery period. Participants were asked to move a cursor in a 2D space in which each axis represent the SMR-ERD in the alpha- and beta-band. During relaxation, participants were asked to move the top right zone where signal strength is increased relative to the previous period. Meanwhile, the during imagery participants were asked to move the bottom left to attenuate the signal strength (i.e., SMR-ERD). Participants were allocated to one of two groups: the verum and placebo groups. While those in the verum group received the online neurofeedback of their EEG, those in the placebo received the yoked-sham neurofeedback, that is the SMR-ERD calculated from the previously acquired data of the other participant^44^.

## Data Records

All datasets were deposited to the OpenNeuro data repository in the EEG-BIDS format^45^ as a separate project^46–49^. The demographic information on participants and relevant information on the allocated group for each participant can be found in the “*pariticpant.tsv*” file. In each directory for a participant, all EEG measurements were saved as the edf format in a block-by-block manner. Links to each dataset are found in the “*readme.md*” file in the github repository (https://github.com/Junichi-Ushiba-Laboratory/pj-hd-smrbmi).

## Technical Validation

**Fig. 2:**
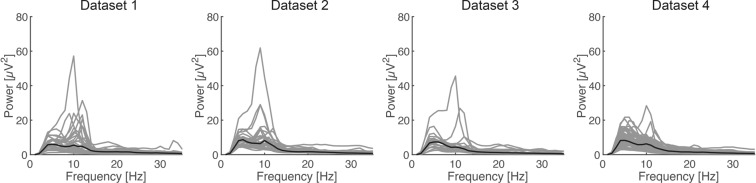
Power spectra derived from four datasets. Gray lines indicate the average value from single participant and black line indicates the global mean. Four panels represent scalp EEG signals derived from C3 channel from each dataset, respectively.

We evaluated the quality of EEG measurement for each dataset. the power spectra derived from SM1 contralateral to the imagined hand (C3 channel) exhibited typical 1/f slopes and a peak at around 10 Hz (Fig. 2). The short-term Fourier transform was applied to the EEG signals using 1 s sliding windows with 90% overlap.

For the qualitative assessment of task-related EEG response, we visualized the event-related spectrum perturbation (ERSP) values from C3 channel. Using the rest period as the reference, ERSP values were calculated using the following formula^22^:

$$ERSP\left(f,t\right), \% =100\times \frac{A(f,t)-R(f)}{R(f)}$$

where *A* is the power at time *t*, in the frequency *f*, and *R* is the reference power calculated as the average of rest period.

**Fig. 3:**
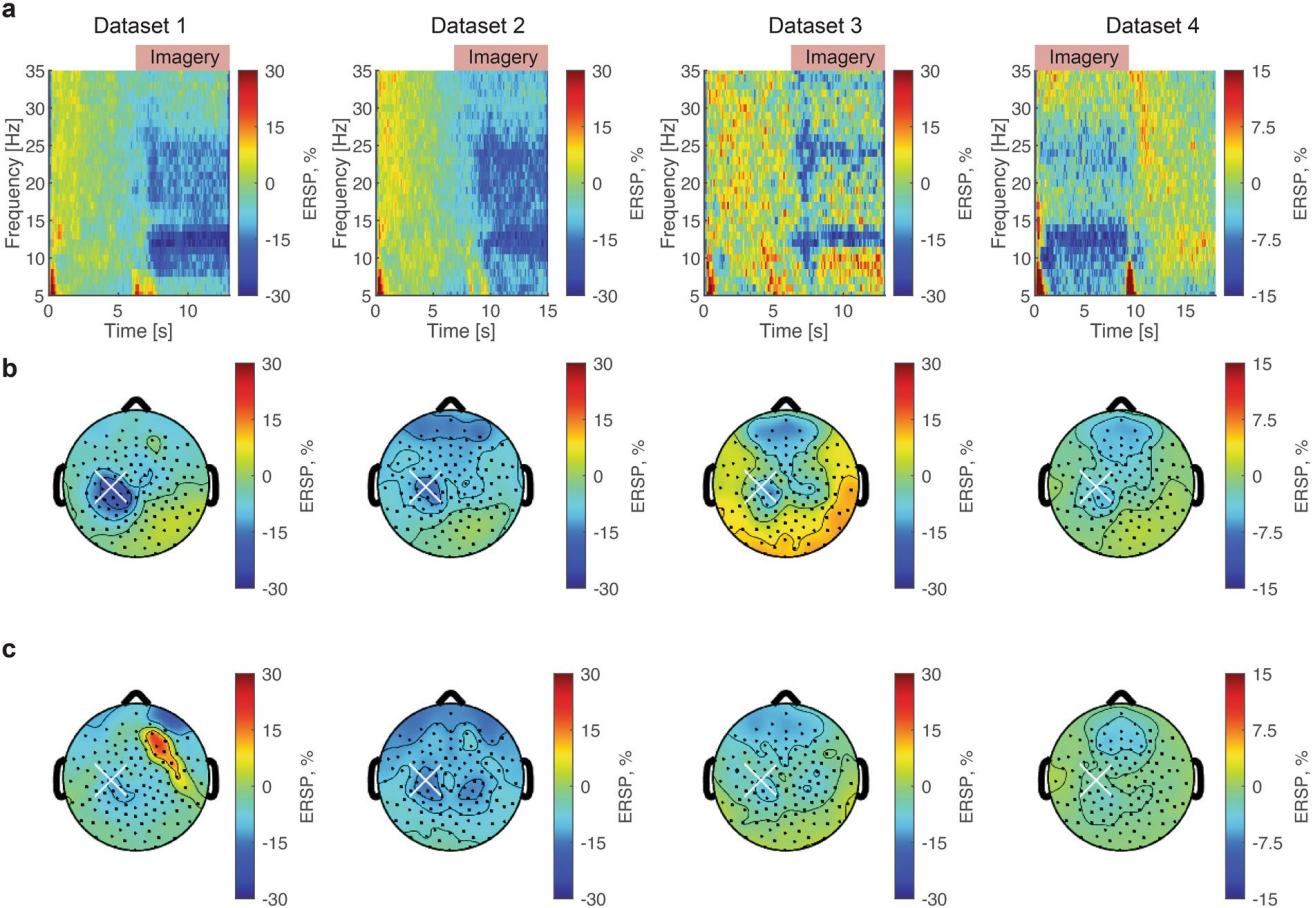
Time-frequency representations and Topographic maps of SMR-ERD from each dataset. (**a**) Time-frequency maps of scalp EEG signals around the sensorimotor cortex contralateral to the right hand (C3 channel). Across datasets, the task-related power attenuation in 8–30 Hz was observed during imagery period. (**b**) Topographic representations of the task-related spectral power change in the alpha-band (8–13 Hz). The white cross indicates C3 channel. (**c**) Topographic representations of the task-related spectral power change in the beta-band (14–30 Hz).

As shown in Fig. 3a, task-related, frequency-specific (8–30 Hz) power attenuation was observed across datasets. The time-specific power attenuation during motor imagery period is consistent with the previous reports of SMR-ERD^22^. The topographic representations during the task period in the alpha-band (8–13 Hz, Fig. 3b) and beta-band (14–30 Hz, Fig. 3c) were found around C3 channels. The spatially localized neural task-related spectral power attenuation around C3 channel during task period is consistent with the previous reports of SMR-ERD induced by the motor imagery of right-hand movement^22^.

**Fig. 4:**
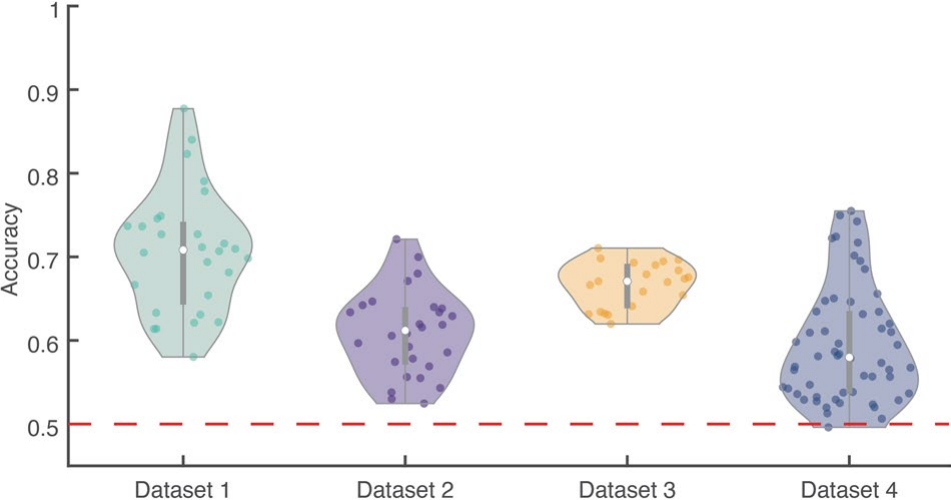
Neural decoding performance using spectral power-based featuresNeural decoding performance to classify the spectral EEG power into the resting-state and motor imagery task was analyzed in a participant-by-participant manner. The distribution of cross-validated accuracy was visualized using the violin plot^54^. The red dashed line indicates the chance-level accuracy.

Finally, we conducted a preliminary analysis of the classification accuracy of resting and imagery period data for each dataset. The accuracy of neural decoding was shown in Fig. 4. In the present analysis, we used the spectral power-based features combined with the random forest algorithm^50^ and conducted one-session leave-out cross validation in a participant-by-participant manner. The individual alpha- and beta-frequencies were identified from the power spectra after subtracting the 1/f component^51,52^.

## Data Availability

Custom codes to reproduce the results of data quality check are available from the GitHub repository (https://github.com/Junichi-Ushiba-Laboratory/pj-hd-smrbmi). To load the edf file format, EEGLAB package and corresponding add-on packages are required^53^. Detailed information can be found in *“readme.md”* at the repository.
